# Microevolutionary dynamics of a macroevolutionary key innovation in a Lepidopteran herbivore

**DOI:** 10.1186/1471-2148-10-60

**Published:** 2010-02-24

**Authors:** Hanna M Heidel-Fischer, Heiko Vogel, David G Heckel, Christopher W Wheat

**Affiliations:** 1 Department of Entomology, Max-Planck-Institute for Chemical Ecology, Beutenberg Campus, Hans-Knoell-Str 8, 07745 Jena, Germany; 2 Department of Biology, 208 Mueller Lab, Pennsylvania State University, University Park, PA, 16802, USA; 3 Department of Biological and Environmental Sciences, University of Helsinki, PL 65, Viikinkaari 1, 00014 Helsinki, Finland; 4 Centre for Ecology and Conservation, School of Biosciences, University of Exeter, Cornwall Campus, Penryn, Cornwall TR10 9EZ, UK

## Abstract

**Background:**

A molecular population genetics understanding is central to the study of ecological and evolutionary functional genomics. Population genetics identifies genetic variation and its distribution within and among populations, it reveals the demographic history of the populations studied, and can provide indirect insights into historical selection dynamics. Here we use this approach to examine the demographic and selective dynamics acting of a candidate gene involved in plant-insect interactions. Previous work documents the macroevolutionary and historical ecological importance of the nitrile-specifier protein (*Nsp*), which facilitated the host shift of Pieridae butterflies onto Brassicales host plants ~80 Myr ago.

**Results:**

Here we assess the microevolutionary dynamics of the *Nsp *gene by studying the within and among-population variation at *Nsp *and reference genes in the butterfly *Pieris rapae *(Small Cabbage White). *Nsp *exhibits unexpectedly high amounts of amino acid polymorphism, unequally distributed across the gene. The vast majority of genetic variation exists within populations, with little to no genetic differentiation among four populations on two continents. A comparison of synonymous and nonsynonymous substitutions in 70 randomly chosen genes among *P. rapae *and its close relative *Pieris brassicae *(Large Cabbage White) finds *Nsp *to have a significantly relaxed functional constraint compared to housekeeping genes. We find strong evidence for a recent population expansion and no role for strong purifying or directional selection upon the *Nsp *gene.

**Conclusions:**

The microevolutionary dynamics of the *Nsp *gene in *P. rapae *are dominated by recent population expansion and variation in functional constraint across the repeated domains of the *Nsp *gene. While the high amounts of amino acid diversity suggest there may be significant functional differences among allelic variants segregating within populations, indirect tests of selection could not conclusively identify a signature of historical selection. The importance of using this information for planning future studies of potential performance and fitness consequences of the observed variation is discussed.

## Background

Studying plant-insect interactions provides an opportunity to investigate the coevolution of species on a molecular, ecological, and evolutionary level. While ecologists are interested in the overall dynamics and interactions between plants and their insect herbivores, biochemical and molecular level studies focus on the genes and gene products that actually interact between these species groups [1]. Ecological and evolutionary functional genomics (EEFG) combines these approaches in an evolutionary framework, integrating the study of gene function and the fitness consequences of genetic variation [2]. A molecular population genetics understanding is central to EEFG study, as it identifies genetic variation and its distribution within and among populations, reveals the demographic history of the populations studied, and can provide indirect insights into historical selection dynamics. Here we use this approach to obtain conclusions regarding the demographic and selective dynamics acting upon a candidate gene involved in plant insect interactions. We then discuss how this understanding is critical to designing future studies of potential fitness consequences due to candidate gene variation.

Our previous research identified a novel gene used by butterflies to detoxify their otherwise toxic host plants, called nitrile-specifier protein (*Nsp*) [3]. Macroevolutionary study indicates that the evolution of *Nsp *was a coevolutionary key innovation in plant insect interactions [4]. In order to extend these insights down to a microevolutionary level where we can eventually directly examine ongoing selection dynamics, here we present the results of a molecular population genetic study of *Nsp *in *Pieris rapae *(small cabbage white) butterflies (Pieridae, Lepidoptera) which feed upon flowering host plants in the Angiosperm order Brassicales.

Brassicales plants present a formidable anti-herbivore defense system, where the enzyme myrosinase upon tissue damage catalyzes the hydrolysis of its glucosinolate substrates to toxic end products [5-7]. Thorough studies of Brassicales plants, most notably on the model species *Arabidopsis thaliana *and relatives, have identified a complex array of molecules involved in this activated chemical defense system [5,8]. A diversity of myrosinases exist in some Brassicales plants [6], which can be accompanied by a variety of cofactors and coenzymes, resulting in the hydrolysis of glucosinolates to variable end products which can influence feeding behavior [9-11]. Additionally, myrosinase concentration in a given plant tissue has been shown to affect herbivore feeding [8,11]. Glucosinolate diversity is also an important factor driving adaptive evolution. Methylthioalkylmalate synthases (*Mam*), encoded by the *Mam *gene cluster, control an early step in the synthesis of glucosinolates and are responsible for a major part of the glucosinolate diversity in the Brassicaceae family by controlling side chain elongation [12-16]. Within the *Mam *gene family, gene duplication, neofunctionalization and positive selection drive biochemical diversification [12]. Recent study documents the increase in glucosinolate complexity along the Brassicales phylogeny, suggesting that chemical defense complexity increased over time [17] (and unpublished data from Wheat et al.)

While our understanding of the plant side of this plant-insect interaction is well developed, we lack a similar depth of knowledge on the insect side. However, the identification of the *Nsp *gene that enables Pieridae butterfly larvae to circumvent the activated chemical defense of Brassicales plants has begun to provide important insights [3]. *Nsp *is expressed in the midgut of the caterpillars and promotes the formation of nitriles rather than toxic isothiocyanates upon the hydrolysis of glucosinolates. *Nsp *is a unique detoxifying gene that shows no homology to any known detoxifying enzyme [18]. Macroevolutionary studies identified *Nsp *as a key biochemical innovation in the Pieridae family, with a single evolutionary origin likely < 10 million years after the appearance of the Brassicales plants (~90 million years ago) which corresponds to a significantly increased speciation rate of Pieridae lineages which feed upon Brassicales [4].

**Figure 1:**
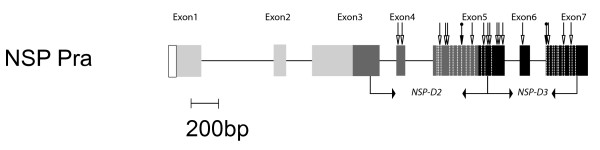
**Structural overview of *Pra Nsp *(EU265817)**. Shaded rectangles and lines respectively represent gene exons and introns to scale. The signal peptide region is indicated by a blank box while the three domains are shaded to different degrees. Depicted are also the approximate annealing sites of the primer pairs used to amplify ~1 kb large segments of the gene and the corresponding names of the fragments. The two segments studied were *Pra Nsp*-D2 (in domain2) and *Pra Nsp*-D3 (in domain3). Dashes show approximate sites of amino acid substitutions located in the amplified coding regions as listed in Fig. 2. The approximate position of selected codons, both positive (black arrow heads with circles on their stems) and negative (open arrow heads), are indicated on exons, with codons listed in Table 4.

*Nsp *has a distinct three-domain structure (Fig. 1) and its enzyme activity is only found in Brassicales feeding Pieridae species [4]. It belongs to an insect specific gene family designated the *Nsp*-like gene family, with members having varying numbers of domain structure repeats [18]. Recent research has found the *Nsp*-like gene family to have complex birth-death dynamics, with *Nsp *paralogs differing in their biochemical activity, genomic location, and copy number within and among species. Additionally, of 5 Pierinae genera surveyed, 4 independent gene duplications of *Nsp*-like genes have been identified. When *Nsp *duplication is placed within the temporal context of increasing glucosinolate complexity of the Brassicales, *Nsp *diversification appears to be an important component of the evolution of this detoxification gene family.

Thus, although previous macroevolutionary study indicates that the first appearance of *Nsp *was a key event in the host shift of pierid butterfly ancestors from Fabaceae to Brassicales feeding [4], and that *Nsp *continued to evolve along with glucosinolate complexity (and unpublished data from Wheat et al.), we know nothing about the modern day, population level dynamics of *Nsp *with respect to the highly variable and complex activated plant defense system of the Brassicales. Here, we begin to address microevolutionary questions by conducting a molecular population genetic study in *P. rapae*, from which we originally identified the *Nsp *gene.

*P. rapae *is a highly abundant species native to Europe with up to four generations per year in temperate zones. A high dispersal ability coupled with feeding on common agricultural plants (e.g. rape seed and cabbage) has enabled it to spread rapidly and successfully colonize Australia, New Zealand and North America within the last 120 years [19-21]. *P. rapae *caterpillars have over 17 reported host plants within the Brassicales, and in particular Brassicacaeae, and thus encounter a high diversity of glucosinolate-myrosinase systems which vary in all the previously discussed components.

Several hypotheses emerge when considering the possible microevolutionary dynamics and patterns of diversity at the *Nsp *gene. For comparative purposes, *Nsp *and a set of reference genes (likely to be experiencing normal purifying selection and reflecting demographic effects) were sequenced from the same individuals: four nuclear coding enzymes, as well as a mitochondrial gene, from ten individuals from each of four populations (Italy, France, Germany, and North America). Additionally, the divergence between *P. rapae *and *P. brassicae *(large cabbage white) among 70 randomly chosen genes was compared with the divergence at *Nsp*. These datasets allow us assess patterns of genetic diversity at *Nsp*, the demographic history of these populations, and the relative support for alternative hypotheses of historical selection at the *P. rapae Nsp *locus (Table 1).

**Table 1:** Alternative hypotheses for the microevolution of *Pra Nsp*.

Hypothesis	Assumption	Expected pattern of variation
H0-demography	No adaptive role	Reflects demographic history
H1-local adaptation	Unique local host plant adaptation = directional selection within populations	Variation within populations < variation among populations
H2- diversifying/balancing selection	Generalist response to diverse host plant assemblages = diversifying/balancing selection	Variation within populations ≥ variation among populations
H3-directional selection	Purifying selection upon optimal genotype = directional selection across populations	Little variation within and among populations

Our alternative hypotheses of selection begin with a working null hypothesis that assumes no historical selective differences among *Nsp *variants, with current patterns of genetic variation at *Nsp *solely reflecting demographic effects such as population structure or historical population expansion (H0-demography). Hypothesis one (H1-local adaptation) expects the *P. rapae Nsp *locus to be involved in local host plant adaptation, showing unique alleles in each population with greater variation among than within populations. Hypothesis two (H2-diversifying/balancing selection) proposes a high diversity of the *P. rapae Nsp *locus across all populations due to *P. rapae *being a highly dispersive generalist, encountering a diverse spectrum of host plants across its range. This hypothesis thus predicts a greater diversity within populations than among them. A further hypothesis (H3-directional selection) assumes low diversity in the *P. rapae Nsp *locus both within and across populations, due to strong purifying selection on the *P. rapae Nsp *locus coupled with selective sweeps since diverging from a recent ancestor.

## Methods

### Biological material

Ten *P. rapae *adults were collected in the wild at each of three different locations in Europe in the summer of 2002. In Germany (DE) samples were taken 1 km north of Jena, in France (FR) from 50 km northeast from Lyon, and in Italy (IT) from 15 km south of Modena. An additional ten *P. rapae *adults were collected in Ithaca, New York, USA (US) in the summer of 2007. Thus, a total of 40 butterflies were kept at -20°C until their DNA was isolated.

### DNA Extraction and PCR

Abdomens of the adult butterflies were homogenized with a TissueLyser (Eppendorf) in the buffer system provided by the genomic DNA extraction kit (Qiagen), and the genomic DNA isolated using genomic tip 20/G columns and the genomic DNA extraction Kit following the manufacturer's protocol (Qiagen). The Eppendorf Master Mix (Eppendorf) was used for the amplification of the desired gene. The PCR products were extracted using a DNA purification kit following the manufacturer's protocol (Zymogen). PCR amplicons were cloned into the pCR II TOPO vector (Invitrogen) with six clones picked per individual and sequenced for all genes with the exception of Arginine KInase and Wingless, where the PCR amplicons were directly sequenced.

### Amplified genes

The study species *P. rapae *only posseses one *Nsp *locus, designated as *Pra Nsp*. Two segments of the *Pra Nsp *gene located adjacent to each other were amplified from genomic DNA, here referred to *Pra Nsp-D2 *and *Pra Nsp-D3 *(Fig. 1). The five reference gene regions studied did not contain introns: isocitrate dehydrogenase (*Idh*), Glyceraldehyde dehydrogenase (*Ga3pdh*), Cytochrome oxidase I (*COI*), Wingless (*Wingless*) and Arginine Kinase (*ArgKinase*).

Primer sequences were as follows in 5' -3' direction: Pra*Nsp-D2*for: tcggctagtcctgctttcaa, Pra*Nsp-D2*rev: tgtgttgtcaagggtgtcca, Pra*Nsp-D3*for: tggacacccttgacaacaca, Pra*Nsp-D3*rev: gtaaagggcaggcacgaagg, Pra*Ga3pdh*for: aaaagggagccaaggttgtt, Pra*Ga3pdh*rev: acgccacaattttcctgaag, Pra*IDH*for: tgctaccatcacaccagatga, Pra*IDH*rev: accaaattcctgcaccttca, *Prawingl *for: acctgttggatgcggctacc, *Prawingl *rev: gcaccgttccactacgaaca, *PraArgK *for: taactgargcycagtacaagga, *PraArgK *rev: gttggtggggcagaaggt

### Sequencing

Plasmid minipreparation from bacterial colonies grown in 96 deep-well plates was performed using the 96 robot plasmid isolation kit (Eppendorf) on a Tecan Evo Freedom 150 robotic platform (Tecan). Each plasmid prep was sequenced in both directions, for a minimum of 2 reads for each clone, of which there were 6 per individual, for 40 individuals, for the two *Nsp *gene regions. This required 960 sequencing reactions for the *Nsp *gene and all reference genes; with the exception of *ArgK *and *Wingless*, for which the amplicons were directly sequenced in both directions without cloning.

Single-pass sequencing of the 5' termini of cDNA libraries was carried out on an ABI 3730 xl automatic DNA sequencer (PE Applied Biosystems). Sequences have been deposited in Genbank under the following Accession numbers (GU215458-GU215936).

### Data analysis

Vector clipping, quality trimming and sequence assembly were done using the Lasergene software package (DNAStar Inc.). The obtained contig assemblies were aligned using the Clustal W [22] program as implemented in the freeware BioEdit program and corrected by eye. Standard measures of DNA polymorphism, demographic analysis and selection, as well as the G-test, were calculated using DnaSP version 4.50.2 [23] including nucleotide diversity (π) [24], nonsysnonymous and silent site substitutions ns/nn [24] within *P. rapae *as well as across species (ω) [25], number of segregating sites (S), theta per site from S (θ; defined as 4Neμ) [25], Tajima's D [26], the McDonald-Kreitman (MK) Test [27] as well as Fay and Wu's H [28] and Fu and Li's D with and without outgroup [29]. For outgroup analysis *Pieris brassicae *sequence information was used. P-values were determined using coalescent simulations (10,000 runs) of a standard neutral model as implemented in DnaSP. Finally, multilocus tests of selection used the maximum-likelihood-ratio Hudson-Kreitman-Aguadé test (ML-HKA-test) [30]. Simulations found that 100,000 chains were sufficient for convergence and the starting value of divergence time for the Markov chain (T) was obtained using a standard HKA test for the reference genes, implemented in DnaSP.

For the following calculations the Arlequin Software package was used [31]. Population genetic structure in *P. rapae *populations was examined using an analysis of molecular variance (AMOVA) [32,33], with samples classified by populations and groups (USA vs Europe) and molecular variation was tested within populations, among populations and between groups. Significance was determined by 10,100 permutations. Population pairwise Fst was estimated by the AMOVA [34]. The significance of the estimated Fst was determined via Markov chain analysis [35] using 10,000 permutations. For Fst estimation, population samples were compared in all pairwise combinations with a sequential Bonferroni adjustment applied to control for Type I errors [36]. Migration rate (m) [37], and from m the absolute number of migrants exchanged between two populations (M), were computed. An exact test for population differentiation was also computed and is equivalent to the Fisher's exact test, which tests the null hypothesis of identical allelic distribution across all populations. Significance was determined via Markov chain analysis with 400,000 steps and 100,000 dememorization steps, again applying Bonferroni adjustment when screening for significant values.

### Demographic history analysis

Approximate Bayesian Computation (ABC) analysis [38] was used to infer the demographic parameters of a simple population expansion model for *P. rapae *as implemented in the software package ABCreg [39]. Given a set of prior demographic parameters used in a coalescence simulation program (ms) to generate population datasets from which summary statistics are calculated, this method uses a linear regression to estimate the posterior distribution of these parameters based upon their similarity to a set of summary statistics obtained from observed data. Our model has three parameters, modern theta (*θ*_0_), the time of the beginning of expansion from refugia (*t*_*b*_), and growth rate of the expansion (*g*). We used a two step approach, beginning with a broad range of prior conditions, which was followed by a more focused range of prior conditions based on the outcome of the first analysis. For both runs, posterior parameter determination was conditional upon θ, π, and R^2 ^from our pooled reference gene dataset (*IDH*, *Ga3pdh*, *Wingless*), which had a minimum of 132 chromosomes sampled. R^2 ^is a statistic that is very sensitive to population expansion [40] and robust to recombination effects [41]. Recombination effects are very important considerations in our dataset. For although other methods for detecting demographic change, such as Fu's Fs and mismatch distributions, are highly significant for our genes and show distributions of pairwise differences consistent with population expansions, these are highly sensitive to recombination effects. Although recombination rates in our reference genes are low, we cannot accurately estimate their upper limits, which then brings these latter results into question. Thus, we have chosen the ABC method to model our demographic changes using summary statistics robust to recombination effects as a conservative approach. *ArgKinase *was excluded from this analysis as it harbors very little genetic variation and appears to be an outlier given its significantly negative Tajima's D values. Pleistocene and post-Pleistocene population size assumptions are based on the likely population size to persist through the Pleistocene and an expansion size that is an order of magnitude larger (but an order smaller, the assumed effective population size of *D. melanogaster *(roughly 1 × 10^6^)).

### Tests for diversifying selection

A comparison between the nonsynonymous (dN) and synonymous (dS) substitutions rates across a gene sample can be used to assess the historical action of positive or negative selection, with dN < dS indicative of purifying selection and dN > dS suggestive of diversifying selection. Given the recent controversy over which methods perform better in detecting negative (purifying) and positive (diversifying) selection at the codon level [42-46], we implemented a counting method (single-likelihood ancestor counting, SLAC), a random effect likelihood (REL) method, a fixed effects likelihood (FEL) method [47], as well as a fixed effects likelihood analysis that only tests for selection along internal (IFEL) branches of the sample phylogeny and is recommended for detecting older selection events in the history of the sample [48]. For population level samples such as the ones we are analyzing here, recombination must be accounted for and incorporated into analyses [49]. We used a genetic algorithm for recombination detection (GARD) method, which shows excellent performance compared to other recombination detection methods [50,51], and used the resulting inferred, non-recombinant partitions for all analyses. All 4 methods are able to utilize these data partitions, as well as DNA substitution models calculated for a given dataset, which we estimated using the Model selection option on the Datamonkey webserver [52]. We used this approach of determining optimal DNA substitution model, testing for recombination, and using the resulting DNA substitution model and partitioned dataset (when recombination was detected) as inputs for the four codon based tests of selection.

### *P. rapae *vs. *P. brassicae *EST comparison

Random sequencing of cDNA libraries made from *P. rapae *and *P. brassicae *gut tissue and the *Pbr Nsp *sequence of *P. brassicae *have been described elsewhere [18]. 2593 unique EST contigs were identified for *P. rapae *from 8153 sequencing reads, while only 973 were found among 2560 reads of *P. brassicae*. The reciprocal best blast hits between each of these two cDNA libraries to the predicted genes of *Bombyx mori *was used to identify homologous genes in both *Pieris *EST collections. A random sample of 70 such homologous pairs was chosen for further analysis. Identified sequences were aligned by Clustal X [22] and each visually inspected for regions of high quality sequence and alignment. End regions of alignments were trimmed such that reading frame (i.e. amino acid sequence) was identical for 3 consecutive codons. Degenerate base pair calls were included. Maximum likelihood estimates of the number of pairwise dN and dS substitutions were performed using codeml of the PAML software package [53], with the estimates of codon frequencies set as free parameters (option F3 × 4). Statistical analyses of dS, dN, and dN/dS (ω) distributions were performed with Jmp 5.0 (SAS Inc.). Non-normal distributions were -log transformed to achieve normality for subsequent determination of significance, but all confidence intervals are reported for the untransformed distributions to keep values in a relevant scale for comparison.

## Results

### Molecular variation

We examined variation in two segments of the *Pra Nsp *gene (*Pra Nsp-D2 *and *Pra Nsp-D3*), covering *Nsp *domains 2 and 3, as well as the exons of five reference genes: isocitrate dehydrogenase (*Idh*), glyceraldehyde dehydrogenase (*Ga3pdh*), arginine kinase (*ArgKinase*), *Wingless *and a portion of the mitochondrially-encoded Cytochrome oxidase I (*COI*) gene. All genes in all populations harbored genetic variation. Nucleotide diversity (π) was roughly 2 to 3 times higher in *Pra Nsp-D2 *compared to the reference genes with the exception of *Wingless *which showed a similar nucleotide diversity to *Pra Nsp-D2*. *Pra Nsp- D3 *π was nearly double the reference genes again with the exception of *Wingess *which exceeds the nucleotide diversity of *Pra Nsp-D3 *(Table 2). θ_W _showed similar patterns of diversity as π. Synonymous diversity (π_ss_) is the highest in *Wingless*, followed by *Nsp-D2 *and *COX*, which have about 50% higher diversity than the rest of the nuclear genes. Nonsynonymous diversity (π_ns_) is highest in *Pra Nsp-D2*, followed by *Pra Nsp-D3*, followed by the reference genes which have much lower levels of amino acid variation (Table 2). *Pra Nsp-D2 *and *Pra Nsp-D3 *have a π_ns_/π_ss _that is over twice that of *Idh *and more than 20 times that of *Ga3pdh *and *Wingless *(Table 2). In total we identified 37 different haplotypes for *Pra Nsp-D2 *and *Pra Nsp-D3 *and 15 different haplotypes for *COX *and *IDH *in all four populations. For *Ga3pdh *we could identify 16 different haplotypes in all populations. *Wingless *and *Arg Kinase *were sequenced directly, therefore we could not distinguish between different haplotypes between individuals for these two loci.

**Table 2:** Summary statistics of *Pieris rapae *genes for individual and grouped populations.

			**coding**							**whole gene**			**non coding**		
					
**Gene**		**n**	**bp**	**πall**	**θall**	**S**	**πss**	**πns**	**ns/ss**	**bp**	**π**	**θ**	**bp**	**πall**	**θall**
		
	**DE**	20		0.0108	0.00902	19	0.02182	0.00769	0.347378		0.02013	0.01823		0.0405	0.03938
***Pra *NSP-**	**FR**	20		0.0093	0.00795	17	0.02121	0.00593	0.275416		0.0164	0.01326		0.02925	0.02542
**D2**	**IT**	20		0.01042	0.00723	15	0.02307	0.00684	0.292393		0.01689	0.01209		0.02858	0.02087
	**US**	20		0.01145	0.00854	18	0.02783	0.00683	0.240799		0.01783	0.01319		0.02951	0.02342
	**total**	80	594	0.01093	0.01054	31	0.02473	0.00703	0.27995	943	0.01722	0.01797	349	0.03159	0.03494
		
	**DE**	20		0.00739	0.00732	12	0.01476	0.00531	0.356045		0.01337	0.01335		0.02735	0.02759
***Pra *NSP-**	**FR**	20		0.0062	0.00671	11	0.01262	0.00437	0.342969		0.01241	0.01335		0.02665	0.02759
**D3**	**IT**	20		0.00562	0.00549	9	0.01159	0.00392	0.335038		0.01247	0.01092		0.02809	0.02519
	**US**	20		0.00538	0.00549	9	0.0153	0.00256	0.16538		0.01114	0.0105		0.02444	0.02269
	**total**	80	462	0.00642	0.00918	21	0.01419	0.00422	0.294649	706	0.01255	0.01537	244	0.02681	0.03279
		
	**DE**	20		0.00459	0.00613	9	0.01723	0.00084	0.048752176						
	**FR**	18		0.00422	0.00492	7	0.01062	0.00233	0.219397363						
**IDH**	**IT**	18		0.00155	0.00281	4	0.00562	0.00035	0.06227758						
	**US**	18		0.00394	0.00524	7	0.01144	0.00174	0.152097902						
	**total**	74	291	0.00365	0.00755	15	0.01163	0.00131	0.112639725						
						
	**DE**	18		0.0042	0.00496	6	0.01564	0.00042	0.02685422						
	**FR**	20		0.00302	0.0016	2	0.01217	0	0						
**Ga3pdh**	**IT**	18		0.00743	0.00743	9	0.01781	0.00042	0.023582257						
	**US**	18		0.00418	0.00248	3	0.01684	0	0						
	**total**	74	352	0.00444	0.00583	10	0.0173	0.00021	0.012138728						
						
	**DE**	8		0.0079	0.00715	14	0.03026	0.00075	0.024785195						
	**FR**	8		0.01003	0.01022	20	0.03865	0.00088	0.022768435						
**COI**	**IT**	8		0.00629	0.00715	14	0.02595	0	0						
	**US**	7		0.00164	0.00162	3	0.03026	0.00075	0.024785195						
	**total**	31	755	0.00691	0.00963	28	0.02611	0.00076	0.029107622						
						
	**DE**	12		0.00321	0.00637	5	0.01246	0.00081	0.064669						
**Arg**	**FR**	20		0.00077	0.00217	2	0.00187	0.00081	0.259259						
**Kinase**	**IT**	20		0.00219	0.00434	4	0.00876	0.00049	0.054933						
	**US**	14		0.00156	0.00242	2	0.0076	0	0						
	**total**	66	260	0.00182	0.00566	7	0.00719	0.00043	0.058823						
						
	**DE**	20		0.01778	0.01603	12	0.06822	0.00127	0.017609						
	**FR**	18		0.01208	0.01378	10	0.04898	0	0						
**Wingless**	**IT**	18		0.01344	0.01102	8	0.05456	0	0						
	**US**	18		0.01159	0.01102	8	0.04487	0.0007	0.015164						
	**total**	74	211	0.01395	0.01556	16	0.05503	0.00051	0.008987						

Shown are the number of sequences (n), the number of base pairs (bp), the average pairwise differences (π), the pairwise differences for synonymous and nonsynonymous sites (π_ss _and π_ns_), θw, the number of segregating sites (S) and the rate of nonsynonymous to synonymous substitutions for the coding part of the genes. If introns are included in the sequence, the number of base pairs, the average pairwise differences and θw is given separately for the whole gene and the non coding part.

**Figure 2:**
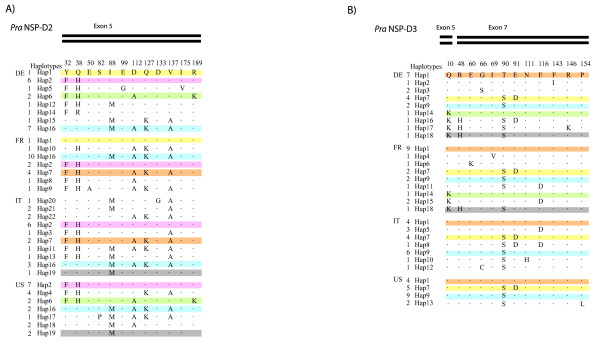
**Overview of the amino acid haplotypes of *Pra Nsp*-D2 (a), *Pra Nsp*-D3 (b) present in each population**. Amino acid variation with reference to first sequence is depicted for each unique allele, with shared alleles across populations highlighted with same color. Bars across the top of the sequences indicates the exon location (Fig. 1).

The location of amino acid polymorphisms varied across the sequenced domains of *Pra Nsp*. Each domain is composed of three exons, with codon lengths of 66, 23 and 112 and 118 respectively (Fig. 1) [18]. The 10 amino acid polymorphisms in *Pra Nsp *domain 2 are only found in its terminal exon (exon five), while 11 of the 14 amino acid polymorphism in *Pra Nsp *domain 3 are also found in its terminal exon; the other three are in the first exon of the domain 3 (exon 5; Figs. 1, 2). The distribution of nonsynonymous polymorphisms across these domains significantly departs from a random distribution based on the size of the exons, with a paucity of amino acid polymorphism observed in the first and second exons, and an excess in the terminal exons, of both domains (G value = 5.99, P = 0.014; Additional file 1 Figure S1). Analyses of synonymous variation does not show such an uneven distribution (Additional file 1 Figure S1). The distribution of synonymous polymorphisms does not show this trend (G value = 0.43, P = 0.512). There was also variation among genes in the number of time a haplotype appeared in a population and in the distribution of haplotypes across populations (Fig. 2). Populations contained both distinct haplotypes as well as some haplotypes that were shared across populations (Fig. 2a, b).

### Population genetic structure

We used AMOVA to partition genetic variance among different levels of population structure for the coding region of all seven gene fragments. Results indicated significant sources of variation within populations for both *Nsp *domains as well as *Idh *and *Ga3pdh*, and among populations within continents for the latter two genes (Table 3). Overall, populations contained the highest percentage of variation compared to variation within and between continents (Table 3). Fst values show an overall low differentiation between populations (Table 4), as most of the variation is located within them (Table 3). After Bonferroni correction of the Fst pvalues we detected significant differences between populations only for comparisons of *Pra Nsp*-D3 between Germany and both France and the USA and in *Ga3pdh *between France and the german, italien and USA populations. *COI *shows Germany and the USA to be differentiated (Table 4). Similarly, across all genes and many population comparisons, the migration rate is high and in many cases indicative of unrestricted gene flow.

**Table 3:** AMOVA results for an estimate of genetic structure.

gene	between continents	among populations	within populations
*NSP-D2*	1.95	5.56	92.49**
*NSP-D3*	7.93	1.43	90.65**
*IDH*	-3.95	10.98**	92.97**
*Ga3pdh*	-10.77	18.66**	92.11**
*COX*	10.84	-0.54	89.7
*ArgKinase*	1.14	0.64	98.22
*Wingless*	-1.61	1.85	99.76

		** P value < 0.01	

Shown is the percentage of variation for each gene at each level of analysis and whether these levels showed significant levels of genetic variation. Comparisons are made within each population and within and between two groups, Europe and the USA.

**Table 4:** Estimates of population differentiation.

Analysis	pop	*Pra *NSP-D2	*Pra *NSP-D3	IDH	Ga3pdh	COI	ArgKinase	Wingless
	DE-FR	0.03	-0,02*	-0.01	0.14*	-0.05	-0.01	0.00
	DE-IT	0.00	0.02	0.03	0.05	-0.03	0.01	0.01
**Fst**	DE-US	0.01	0,09*	-0.02	0.04	0.20*	-0.04	0.00
	FR-IT	0.03	0.04	0.10	0.29**	0.00	0.01	0.03
	FR-US	0.06	0.13	-0.04	0.17*	0.14	0.04	0.00
	IT-US	0.03	0.07	0.07	0.02	0.11	0.05	0.02

	DE-FR	0.0009*	0.0082*	0.4361	0.0279	0.4868	0.0867	0.7204
	DE-IT	0.0321	0.1859	0.3974	0.1286	0.5966	0.4368	0.3702
**Exact Test**	DE-US	0.0373	0.0001*	0.9271	0.0177	0.0507	0.6965	0.1252
	FR-IT	0.1009	0.0911	0.0060	0.0022*	0.1747	0.6628	0.4554
	FR-US	0.0007*	0.1322	0.7671	0.0525	0.3480	0.1460	0.2003
	IT-US	0.0039	0.1107	0.0842	0.5326	0.2481	0.2489	0.0310

	DE-FR	15.48	**inf**	**inf**	3.01	**inf**	**inf**	**128.35**
	DE-IT	**101.85**	**30.21**	14.32	10.31	**inf**	**90.30**	**35.16**
**Migration**	DE-US	**45.25**	4.92	**inf**	12.93	1.93	**inf**	**inf**
	FR-IT	16.88	11.76	4.65	1.22	**inf**	**33.18**	16.44
	FR-US	8.20	3.33	**inf**	2.40	3.04	10.70	**inf**
	IT-US	15.79	6.49	6.50	**32.73**	4.23	10.06	20.90

Fst values, p-values for the exact test and the estimated absolute number of migrants between two populations M as determined in the Arlequin program are given for every population comparison for every sequenced gene. For easier comprehension high migration values (above 30) are in bold. Analysis always includes the whole sequenced fragments, thus both exons and intron in *Pra Nsp*-D2 and -D3. For both the Fst and Exact Tests, P-values significant after Bonferroni corrections (< 0.05) within gene are marked with an asterisk. If the p-value is < 0.001 (after Bonferroni) two asterisks are used.

Exact tests for population differentiation reveal roughly the same low level population structure observed in the Fst analysis (Table 4). First, analysis of *Nsp*-D3 finds the same pairwise comparisons significant as in the Fst analysis. This is also observed at *Ga3pdh*, although only the largest of the three significant Fst comparisons is significant in the exact tests. Second, breaking with this pattern is *Nsp*-D2, where only the extact test finds differences between France and both Germany and the USA. While Fst analyses use the number of genetic differences between haplotypes to assess structure, the exact test uses the haplotype identities themselves and is thus more sensitive to recombinant haplotypes. Thus these observations likely derive from differences in the distribution of recombinant haplotypes and thus indicate population structure at a much finer scale than detected in the Fst analysis. Third, it is important to note that there is little if any population structure found across all of the reference loci (Table 4).

### ABC analysis of demography

Our investigation of the demographic history of these samples began with determining the posterior distribution of demographic parameters for a broad set of prior conditions. Given the Quaternary history of Europe and the phylogeographic structure of many species [54], we estimate the mid-Pleistocene Ne of *P. rapae *to be 10,000 which expanded to a modern size of 100,000. In our first run we tested this hypothesis by drawing prior conditions from a wide uniform distribution where the onset of population expansion, t_*b*_, was between 0 and 100,000 years ago. Population growth rate, *g*, assuming an expansion to a modern N_0 _of 100,000 from a size of 10,000 is 11.5. With this in mind, we drew priors from a broad uniform distribution around this ideal, with *g *ranging from 0 to 20. A liberal tolerance for acceptance (0.01 of priors) was used to screen through 1,000,000 prior simulations, with acceptance contingent upon similarity to a set of summary statistics (θ, π, and R^2^) from our reference gene dataset (*Wingless*, *IDH*, *Ga3pdh*). This first run returned a mean *g *of 1.7 (lower and upper values = 1.35 - 2.31) and t_*b *_of 7,881 generations before present (2,947 - 13,692). Our second run used a more narrow acceptance criteria (0.001) and a more focused range of prior values based upon 2 times above and below the observed means from the first run (prior ranges: *g *= 0 - 5.103; t_*b *_= 0 - 24,000 generations). Second run results returned posterior estimates of mean *g *being 2.85 (lower and upper values = 0.72 - 4.27) and t_*b *_being 9,420 generations before present (3,717 - 18,102).

### Tests for selection

We employed molecular tests of selection based on the null hypothesis of the standard neutral model. Tajima's D is not significant for any of the tested gene regions with the exception of the German population sample of *ArgKinase *(Tajima's D = -1.83, P < 0.05). The most positive values of Tajima's D are found in *Pra Nsp-D2 *while all the other genes have negative values or are close to zero (Additional file 1 Table S1). Tajima's D for *Nsp-D2 and -D3 combined *was -0.44. Fu and Li's D also found no significant genes other than *ArgKinase *in the German population (Fu and Li's D = -2.23, P < 0.05), either with or without *P. brassicae *as an outgroup (Additional file 1 Table S1). Analysis of the relationship of non-synonymous vs. synonymous polymorphism within species to non-synonymous vs. synonymous divergence between species used the McDonald-Kreitman (MK) test with *P. brassicae *as an outgroup (Additional file 1 Table S2). Results for all genes are not significant, although the number of nonsynonymous fixed substitutions was highest in *Pra Nsp *(n = 48). This is more than an order of magnitude higher than the next highest reference gene (n = 3, *Idh*), while *COI *had the highest number of synonymous substitutions (n = 73) followed by *Nsp *(n = 45; Additional file 1 Table S2). Removing low frequency haplotypes in the *Nsp *datasets, with an occurrence of two or less, also results in non-significant MK tests (not shown). The multilocus HKA tests on either of the *Pra Nsp *regions (*Pra Nsp-D2 *and *Pra Nsp-D3*), tested individually against the reference genes and in combination, showed no significant divergence from the neutral expectations. Analyses conducted on pooled population samples also found no significant departures from neutral expectations (results not shown).

We also implemented molecular tests of selection that were focused on detecting diversifying selection at the codon level, in the presence of recombination, while making no assumptions about the demographic history of the underlying sample.

Genetic algorithm analysis detected a recombination breakpoint (P < 0.01) at bp 264 in the *Pra Nsp-D2 *gene dataset, but not in other datasets. Therefore a partitioned dataset of the *Pra Nsp-D2 *dataset was used in all subsequent analyses (provided as output from the GARD analysis and available in Additional file 1 GARD tree file). Previous simulation study of the Type I and Type II error rates of the SLAC, FEL, and REL methods recommends using a P-value cutoff of 0.25 for the first two methods and a focus upon sites that are identified as under selection by more than one method [47]. Focusing upon sites that have a P-value less than 0.15 and are shared by at least two of these three different methods, we found one positively selected site in both *Nsp *domain 2 and 3, as well as many negatively selected sites in both domains (Fig. 1, Table 5). Of these three methods (SLAC, FEL, and REL), SLAC returned the least significant P-values while the posterior probabilities of the REL method are usually very significant. The IFEL method identified several sites that have changed in selection pressures over evolutionary time, identifying negatively selected sites in both domains (Table 5), as well as the positively selected site in *Nsp *domain 2 (codon 112, P = 0.021). No similar evidence for positive selection was found in the other genes. However, all genes showed evidence for negatively selected sites (i.e. sites under purifying selection), ranging from 1 in arginine kinase to 21 in cytochrome oxidase I (*COI*).

**Table 5:** Selected sites in *P. rapae *NSP domains 2 and 3 identified by at least two of the methods

			Analysis Method			
			
Codon	Domain	**Sel**.	SLAC	FEL	IFEL	REL
276	2, exon 4		-37.08 (0.246)	-24.60 (0.236)	-24.60 (0.328)	**-5.28 (3348.03; 0.001)**
277	2, exon 4	**N**	**-136.86 (0.004)**	**-120.18 (0.002)**	**-120.18 (0.010)**	**-17.83 (1,207,160,000.00; < 0.001)**
284	2, exon 5		-30.24 (0.302)	-22.12 (0.195)	-22.12 (0.280)	**-1.36 (95.85; 0.029)**
287	2, exon 5	**N**	-27.37 (0.333)	**-59.18 (0.071)**	**-59.17 (0.115)**	**-13.32 (31,730.30; 0.001)**
299	2, exon 5		12.69 (0.745)	13.36 (0.358)	23.00 (0.257)	**8.57 (260.85; 0.0013)**
303	2, exon 5		-37.08 (0.246)	-26.42 (0.224)	-26.41 (0.313)	**-6.12 (3,645.68; 0.001)**
316	2, exon 5	**N**	**-93.32 (0.109)**	**-340.07 (0.020)**	**-340.07 (0.035)**	**-17.06 (49,267.90; 0.001)**
329	2, exon 5	**N**	-46.91 (0.242)	**-81.89 (0.070)**	-81.89 (0.136)	**-8.79 (2,163.10; 0.002)**
334	2, exon 5	**N**	**-111.25 (0.015)**	**-85.83 (0.006)**	**-85.82 (0.019)**	**-17.82 (55,812,800.00; < 0.001)**
338	2, exon 5		-27.37 (0.333)	-20.84 (0.181)	-20.83 (0.263)	**-1.31 (93.12; 0.03)**
355	2, exon 5		22.27 (0.672)	29.94 (0.259)	50.70 (0.163)	**8.53 (114.69; 0.0031)**
379	2, exon 5	**P**	28.03 (0.488)	**63.06 (0.108)**	**201 (0.021)**	**8.64 (2,410.59; < 0.001)**
389	2, exon 5		-29.36 (0.333)	-33.44 (0.161)	-33.44 (0.300)	**-1.31 (93.37; 0.03)**
390	2, exon 5	**N**	-29.36 (0.333)	**-92.94 (0.087)**	-92.95 (0.186)	**-3.76 (143.98; 0.02)**
393	2, exon 5		-16.89 (0.579)	-35.67 (0.201)	-35.68 (0.355)	**-1.30 (93.64; 0.03)**
395	2, exon 5		-29.36 (0.333)	-32.64 (0.181)	-32.64 (0.329)	**-1.30 (92.46; 0.03)**
405	2, exon 5		-29.36 (0.333)	-32.54 (0.213)	-32.54 (0.369)	**-1.30 (92.02; 0.031)**
430	3, exon 5	**N**	-39.78 (0.246)	**-40.08 (0.123)**	-40.08 (0.244)	**-1.55 (103.69; 0.027)**
434	3, exon 5		-32.44 (0.302)	-35.73 (0.192)	-35.73 (0.342)	**-1.38 (96.44; 0.029)**
437	3, exon 5	**N**	**-102.62 (0.109)**	**-585.52 (0.019)**	**-585.52 (0.046)**	**-11.21 (344.05; 0.009)**
441	3, exon 5	**N**	**-119.35 (0.015)**	**-161.93 (0.021)**	**-161.93 (0.086)**	**-17.73 (830,226.00; 0.001)**
467	3, exon 5		22.31 (0.508)	30.17 (0.245)	36.57 (0.244)	**3.11 (333.06; <0.001)**
480	3, exon 5	**N**	**-96.13 (0.004)**	**-124.06 (0.001)**	**-124.08 (0.0114)**	**-37.22 (30,456.40; 0.0001)**
483	3, exon 6	**N**	-19.22 (0.333)	**-22.54 (0.132)**	-22.54 (0.2689)	**-2.80 (174.20; 0.032)**
493	3, exon 6	**N**	**-38.45 (0.111)**	**-54.50 (0.026)**	**-54.50 (0.0975)**	**-6.97 (194.36; 0.028)**
505	3, exon 7		19.00 (0.455)	19.49 (0.204)	24.46 (0.203)	**3.19 (845.98; <0.001)**
508	3, exon 7	**N**	**-38.45 (0.111)**	**-56.79 (0.023)**	**-56.79 (0.0909)**	**-7.59 (201.55; 0.027)**
523	3, exon 7	**P**	28.57 (0.306)	**28.63 (0.122)**	n/a	**3.20 (12725.90; <0.001)**
532	3, exon 7	**N**	-19.22 (0.333)	**-26.85 (0.114)**	-26.85 (0.2402)	**-3.05 (174.17; 0.032)**
548	3, exon 7		16.83 (0.635)	24.43 (0.372)	63.24 (0.181)	**3.05 (114.56; 0.001)**
573	3, exon 7		16.84 (0.636)	24.32 (0.388)	30.54 (0.363)	**3.04 (98.82; 0.001)**
580	3, exon 7	**N**	**-184.42 (0.000)**	**-239.57 (< 0.001)**	**-239.57 (0.0021)**	**-37.41 (1,829,600.00; <0.001)**
594	3, exon 7	**N**	-19.22 (0.333)	**-27.67 (0.110)**	-27.67 (0.2337)	**-3.06 (175.61; 0.031)**

Note. - For every method, first value is scaled dN - dS and number in parentheses shows P value, except for the REL method, where first value is Bayes factor value followed by P value based on the Bayes factor posterior probability). Significant values (P < 0.15) shown in bold and when 2 of the four methods are significant, the Selection column indicates "N" for negative or "P" for positive selection. Codon numbering is relative to start codon of the signal peptide.

### Interspecific divergence and dN/dS

*P. rapae *and its congener *P. brassicae *diverged approximately 11.75 Myr ago, based on temporal calibration of sequence divergence in the EF-1α gene as previously applied to Pieridae [4]. To compare the pattern of divergence at *Nsp *with a random genomic sample of genes, 70 homologous gene pairs were identified in EST collections of these two species. These ranged from a length of 183 to 792 bp, with a mean of 520.9 bps (std. dev. = 144) and 75% of sequences being > 430 bp long. This translates into a mean of 130 synonymous and 390 nonsynonymous sites per gene pair respectively (std. dev. 40.7 and 108 respectively). There was a range of between 5 to 71 bp differences between sequence pairs, with a mean of 27.2 bp (std. dev. = 13.2 bp).

Maximum-likelihood analysis of dS and dN divergence between these *Pieris *species across these 70 genes finds substantial divergence, with the average dS = 0.189 (std. dev. = 0.073) and dN = 0.018 (std. dev. 0.018). However, these genes are, as expected, experiencing a fair amount of purifying selection with a mean dN/dS (ω) = 0.097 (std. dev. = 0.091), with a range from 0 to 0.38.

**Figure 3:**
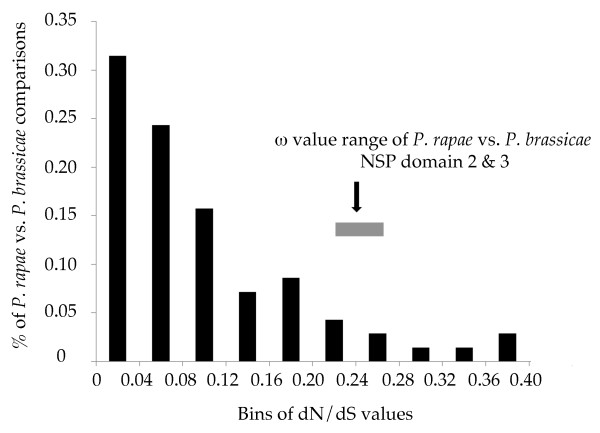
**Frequency distribution of dN/dS ratios (x-axis) from interspecific comparisons among 70 random genes between *Pieris rapae *and *P. brassicae***. The number of genes in each bin of a given ratio range are shown on the y-axis. The range of dN/dS across all pairwise comparisons of *Pra Nsp *domain 2 & 3 combined with *Pbr Nsp *domain 2 & 3 sequence is shown right of the histogram as a grey box. Analyses of domains 2 & 3 separately showed little difference from the combined analysis, and thus the latter was presented for greater figure clarity.

The divergence and ω values at *Nsp *between these species are significantly greater than the 95% confidence interval of the observed genomic mean estimate from the 70 randomly chosen genes. The mean dS and dN across *Nsp *domains 2 & 3 is dS = 0.269 (std. dev. = 0.010) and dN = 0.071 (std. dev. = 0.002). Their range (dS: 0.26 - 0.31; dN: 0.07 - 0.8) is greater than the 95% confidence intervals for the dS and dN values of the random genes (dS: 0.176 - 0.213; dN: 0.016 - 0.025). The combined *Nsp *domains 2 & 3 have a mean ω = 0.25 and the full range of their pairwise values (0.22 - 0.27) is significantly greater than the 95% confidence interval of ω for the 70 homologous genes (0.087 - 0.133; Fig. 3).

## Discussion

Our interest in the *Nsp *gene originates from its central role in hostplant detoxification and the macroevolutionary consequences of this function [3,4]. Like many evolutionary ecology studies that have identified a candidate gene with ecologically relevant function, we wish to know more about the microevolutionary dynamics of its genetic variation. By focusing at the population level within one species, we have conducted a battery of analyses to help discriminate among alternative adaptive hypotheses and uncover the segregating genetic variation upon which future ecological studies should focus (Table 1).

Four alternative hypotheses were developed to assess the microevolutionary dynamics at *Pra Nsp *in the sampled *P. rapae *populations (Table 1). Two of these can be soundly rejected based on our results. First, H1 posits that local adaptation causes a greater level of genetic diversity among than within populations. This hypothesis is rejected by the high diversity of *Pra Nsp *amino acid alleles in all populations and with many alleles shared among populations (Fig. 2). Formally, the *Pra Nsp *loci have low Fst values and high migration rates (Table 4), non-significant Tajima's D and related tests (Additional file 1 Table S1), and AMOVA results that indicated greater variation within than among populations (Table 3). While the exact tests of population differentiation in both *Pra Nsp*-D2 and D3 do give some hint at population structure (Table 4), this test is sensitive to the unique recombinant haplotypes found in populations that are at low frequency (e.g. Fig. 2). Thus, H1-local adaptation is rejected. Second, H3 posited directional selection or strong purifying selection upon an optimal genotype, both of which would result in little variation within and among populations. The high levels of within population amino acid polymorphism at *Pra Nsp *(Fig. 2), as well as the non-significant MK tests (Additional file 1 Table S2), argue conclusively against H3.

The results for the two remaining hypotheses, H0-demography and H2-diversifying/balancing selection, are more complicated. Much of the last decade has been focused upon developing methods to tease apart the effects of selection from demography on the patterns of DNA within populations (the site frequency spectrum). While in some cases this is possible in model genomic species, for non-model species with limited genomic insight, teasing these relative effects apart is very difficult. This is especially true for the case of potential diversifying/balancing selection where molecular tests of selection have very low power [55,56]. While the AMOVA results are consistent with H2-diversifying selection, where *Pra Nsp *diversity is expected to be higher within than among populations (Table 3), these patterns are also seen at two of our reference genes (*Idh *and *Ga3pdh*), the latter of which also shows significant exact test and Fst results in pairwise population comparisons. This suggests that much if not all of our genetic variation is influenced by the recent demographic history of *P. rapae*.

Our null hypothesis, H0-demography, expected genetic variation within and among samples at the reference genes to solely reflect demographic history. Young *P. rapae *females are known to migrate long distances before egg laying [19] and this natural dispersal ability is likely augmented and scrambled due to the long-distance transport of crop plants bearing eggs and larvae [57]. In accordance with the high dispersal of *P. rapae *we find a general pattern of greater genetic diversity within vs. among populations in all genes (Table 3). In addition, the North American sample shows no clear distinction from the European populations, which may be indicative of recent and ongoing movement of *P. rapae *into the Americas instead of one historical introduction. Modeling of the demographic history based solely upon our reference genes indicates a population expansion 9,420 generations before present. The mean to lower range of our estimates (3,717 - 18,102) are consistent with the demographic history of most species in Europe [54] and the known expansion of this species [20,21]. Thus, the patterns of molecular variation at *Pra Nsp *suggest that H0-demography cannot be rejected; *Nsp *genetic variation is strongly influenced by recent population expansion.

One means of circumventing the confounding effects of demography and selection on the site frequency spectrum is to use analyses that are robust to demographic history. We implemented two such tests. First, we used the MK test and found no significant results (Additional file 1 Table S2). This is entirely consistent with the absence of directional selection within our populations (i.e. a rejection of H3). In the context of H2- diversifying/balancing selection, the MK test is not an appropriate test. Hughes [58] has argued that tests of neutrality, and the MK test in particular, only provide an appropriate test for very specific types of selection. Stated another way, no single test is a general test for all types of selection. Thus, what the MK test results tell us is that there does not appear to be an excess of repeated selective sweeps at different codons in any of our genes, since *P. rapae *diverged from their common ancestor with *P. brassicae *(i.e. rejection of H3).

Our second test was a codon based test of selection that looked for both positive (diversifying) and negative (purifying) selection while making no assumptions regarding demographic history and incorporating recombination effects [47]. Such tests have recently been the focus of a vigorous debate in the literature regarding their assumptions and relative performance [42-47], and whether such methods are fundamentally flawed [59]. Thus, rather than picking among these methods we cautiously employed several codon based tests of selection which covered the range of fundamental methodological assumptions, presented a full disclosure of their findings, and identified only codons which found some support across these methods (Table 5). This approach avoids the possible false positives inherent in any one method, but does not get around the multiple testing issues and other problems inherent to these methods [59].

This approach does find evidence in the *Pr Nsp *domains for both negative (purifying) selection and positive (diversifying) selection (Fig. 1, Table 5). While a discussion of the fundamentally different assumptions employed by these methods is beyond the scope of this paper, they are known to differ in their sensitivity and false positive rate [47]. While there is certainly purifying selection acting on certain regions of the *Nsp *gene, the findings of positive selection should be viewed with caution and are not conclusive enough to warrant rejection of H0-demography in favor of H2-diversifying/balancing selection. Ultimately, determination of the evolutionary consequence of any of the observed amino acid variation necessitates functional assessment.

Our final consideration focuses upon the amount and distribution of amino acid diversity within *Pra Nsp*, which cannot solely be accounted for by demographic effects alone. The observed number of amino acid polymorphisms in *Prap Nsp *are greater than the well studied *Pgi *gene in *Colias *butterflies, which may be the most diverse gene known from Insecta in having 15 segregating amino acid sites spread across 556 codons [60]. Combining the information we have for *Pra Nsp *domains 2 and 3, we have identified 24 segregating amino acid polymorphisms across 346 codons (Fig. 1). Considering that we have not even surveyed the first domain of *Pra Nsp*, it is very likely that the *Pra Nsp *gene could harbor over 30 amino acid polymorphisms within populations across its 618 codons (a level that is twice that seen at *Pgi *in *Colias*). In sum, *Nsp *appears to be one of the most polymorphic coding genes known in Insecta. Higher polymorphic levels can be found in the sex-determination gene, complementary sex determination, of honey bees that exhibits trans specific balancing selection [61].

Some of the increased amino acid diversity is certainly due to relaxation of purifying selection at specific regions of the enzyme. There is a well documented gradient of increasing amino acid diversity and divergence with greater solvent exposure of codons in enzyme structures (e.g. [62,63]). This arises due to strong functional constraints on the folding of the enzyme, which is relaxed in enzyme surface regions. Comparing the dN/dS value of *P. rapae Nsp *vs *P. brassicae Nsp *with an average of 70 randomly chosen genes between those two species shows that *Nsp *has a significantly higher dN/dS ratio and more divergence. Given that we were only using a consensus sequence derived from a small number of individuals, we have likely inflated our estimations of divergence with polymorphic differences. Such inflation makes our comparison with *Nsp *divergence more conservative. However, this set of 70 genes, given their shared identification from separate EST libraries, is likely to be enriched for genes having a moderate to high level of expression even though the libraries were normalized. As such, this set of genes likely represents a biased set of genes having housekeeping functions and experiencing moderate to strong purifying selection. Thus, the observation of *Nsp *having a higher dN/dS ratio than these genes only tells us that *Nsp *is under less constraint compared to 70 random housekeeping genes. Importantly, this reduced constraint is not large as NSP only shows a 0.2 higher dN/dS ratio than the gene average, indicating ongoing purifying selection for the gene function. However these values are gene averages, therefore a more detailed assessment is needed.

Detailed examination of the distribution of amino acid variation across the sequenced domain regions shows functional constraint acting on specific regions of the enzyme coupled with an unexpectedly high amount of amino acid polymorphism concentrated in specific domain regions (Fig. 1, Table 5, Additional file 1 Figure S1). Although further study is necessary to fully document this observation, as data from the first domain and all of the second domain are needed; such patterns indicate substantial variation in functional constraint across the gene. These results suggest that greater knowledge of the structure-function relationships of the *Nsp *protein will be necessary in order to understand the observed excess amino acid variation. In sum, while regions of relaxed constraint certainly harbor more variation, this does not mean such variation is neutral. Indeed, much of the known amino acid variation affecting the kinetics of metabolic enzymes is located upon the surface of enzymes (e.g. [60,62-64]).

## Conclusion

The microevolutionary dynamics at the *Nsp *gene appear to be a mixture of demographic effects (population expansion and high migration) coupled with variation in functional constraint across the gene. Patterns of nucleotide diversity and indirect molecular tests for historical selection reject strong local adaptation, as well as directional and strong purifying selection. Rather than taking the absence of clear signatures of historical selection upon the *Nsp *gene as conclusive evidence for no fitness variation among alleles, we recognize the limitation of such indirect approaches and remain curious as to the functional effects of the extremely high amount of amino acid diversity. Thus, this study provides a foundation for the design and insightful use of molecular markers for genetic variants whose ecological performance and fitness can be characterized in the field (e.g. [65]).

We now know that there is an exceptional amount of amino acid variation within nearly every population of *P. rapae*. If this allelic variation has functional consequences, the effects are likely to be environmentally dependent and potentially small. As such, future studies will need very large sample sizes for many families across a range of potentially relevant conditions. Families can be sampled from the field as ovipositing females and will contain sufficient diversity for study. In addition, individuals will need to be sampled during larval stages in order to provide access to the cDNA of the *Nsp *gene, as the entire coding sequence must be sequenced for no single polymorphic site will suffice to characterize *Nsp *allelic variation. Only with functional study of the identified genetic variation can we begin to conclusively assess the extent to which the observed variation at *Nsp *plays an ongoing role in the microevolutionary dynamics of *P. rapae *and its interaction with the highly variable chemical defense system of their Brassicales hostplants.

## Authors' contributions

HH-F carried out the molecular laboratory work, with the help of HV. HH-F and CW analyzed the raw data and performed the statistical analyses, CW conducted the ABC analysis. HH-F and CW designed the study and wrote the manuscript. HV and DH participated in its design and helped draft the manuscript. All authors participated in the writing and approved the final version.

## Supplementary Material

Additional file 1 **Additional Figures and Tables**. Figure S1: Comparison of synonymous and nonsynonymous site changes in the NSP domains; Table S1: Summary statistics for molecular tests of selection; Table S2: Summary statistics for MK test; Tree file from GARD output; *Pieris *species cDNA comparison datatable for dN/dS analysis. Click here for file
