# Supplementary material for: Microevolutionary dynamics of a macroevolutionary key innovation in a Lepidopteran herbivore
Source: BMC Evol Biol. 2010 Feb 24;10:60. doi: 10.1186/1471-2148-10-60 (PMC2841170; doi:10.1186/1471-2148-10-60)
Supplement: Additional file 1 — Additional Figures and Tables. Figure S1: Comparison of synonymous and nonsynonymous site changes in the NSP domains; Table S1: Summary statistics for molecular tests of selection; Table S2: Summary statistics for MK test; Tree file from GARD output; Pieris species cDNA comparison datatable for dN/dS analysis. [file 1471-2148-10-60-S1.PDF]

Comparison of synonymous and non-synonymous site change across the NSP domains.

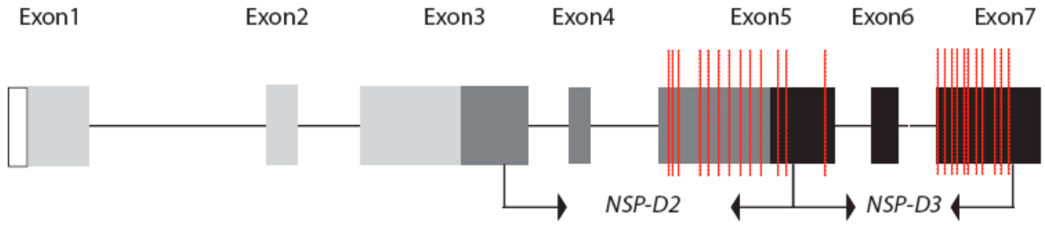

Exon	bps seq'd	codons seq'd	ss changes	ns changes
Domain 2 - exon 3	21	7	0	0
Domain 2 - exon 4	70	23	4	0
Domain 2 - exon 5	356	119	10	10
Domain 3 - exon 5	198	66	5	3
Domain 3 - exon 6	70	23	2	0
Domain 3 - exon 7	323	108	6	11
sum	1038	346		

totals	bps seq'd	codons seq'd	ss changes	ns changes
1st exon of Domains 2 & 3	219	73		
2nd exon of Domains 2 & 3	140	46		
1st + 2nd exons of Domains 2 & 3	359	119	11	3
3rd exon of Domains 2 & 3	679	227	16	21

Analysis by bp: ns changes

====== Input Tabla ======

3	21	|	24
359	679	|	1038
362	700	|	1062

======== Output ========

G-test

G value: 5.994

P-value: 0.01435*

G with Williams' correction: 5.854

P-value: 0.01554*

G with Yates' correction: 4.777

P-value: 0.02884*

Analysis by bp: ss changes

====== Input Tabla ======

11	16	|	27
359	679	|	1038
370	695	|	1065

======== Output ========

G-test

G value: 0.430

P-value: 0.51215 (not significant)

G with Williams' correction: 0.421

P-value: 0.51653 (not significant)

G with Yates' correction: 0.206

P-value: 0.65006 (not significant)

as implemented in DNAsp

Table 1: Summary statistics for molecular tests for selection. Tests marked with an asterix (*)had a value of $P < 0.05$ under the standard neutral model. Tests employing an outgroup are indicated w out.

		Taj D	Fu &Li D	Fu &Li D w out	Fay & Wu H w out
**NSP-D2**	**DE**	0,52466	-0,01758	0,45161	-1,07368
	**FR**	0,62481	0,3006	0,63641	-1,95238
	**IT**	1,65115	0,84646	0,90748	-0,73684
	**US**	1,02003	1,01419	1,06926	-1,88421
**NSP-D3**	**DE**	0,0354	0,22999	0,18187	-0,01053
	**FR**	-0,27156	-0,3242	-0,45619	-0,58947
	**IT**	0,0785	-0,68651	-0,47456	-0,48421
	**US**	-0,07133	0,86241	0,9011	-0,71579
**IDH**	**DE**	-1,13975	-0,49086	0,9011	-3,29474
	**FR**	-0,48363	0,09399	0,02404	0,54902
	**IT**	-1,34736	-0,70114	0,16108	-1,30719
	**US**	-0,89286	-0,97212	-1,21763	0,2381
**Ga3pd**	**DE**	-1,26827	-0,84169	0,20307	-0,8366
	**FR**	0,68713	1,00649	1,01226	-0,30526
	**IT**	-1,53674	-1,7989	-1,64146	-1,33333
	**US**	0,79344	1,1232	1,14969	0,88889
**COI**	**DE**	0,53266	0,73372	1,08928	-2,21429
	**FR**	-0,3401	-0,21328	0,64362	-7,64286
	**IT**	-0,61245	-0,07256	0,09829	-5,28571
	**US**	0,05031	0,38925	0,23258	-0,19048
**ArgKin**	**DE**	-1.83094*	-2.22902*	-1,55257	-1,21212
	**FR**	-1,51284	-2,05308	-2,17757	0,18947
	**IT**	-1,43544	-1,69308	-1,88116	0,50526
	**US**	-0,95919	-0,44573	0,67726	0,21978
**Wingless**	**DE**	-0,17954	-0,66718	-0,86934	2,29474
	**FR**	-0,44384	-0,42276	-0,57994	1,09804
	**IT**	-0,08764	0,49467	0,48159	0,94118
	**US**	-0,23317	-0,11452	-0,21967	1,41176

Table 2: Summary statistics for the McDonald-Kreitman Test as implemented in DNA SP. Significance is tested by the Fisher's exact test,

Gene	Substitution	fixed	polymorphic	Fisher's exact test
**NSP-D2**	syn	29	18	0,641663
	nonsyn	24	11	
**NSP-D3**	syn	16	9	1
	nonsyn	24	13	
**IDH**	syn	11	13	1
	nonsyn	3	4	
**Ga3pdh**	syn	7	10	0,508772
	nonsyn	0	2	
**COI**	syn	73	10	0,181992
	nonsyn	0	2	
**ArgKin**	syn	4	6	1
	nonsyn	0	1	
**Wingless**	syn	7	17	0,545299
	nonsyn	0	3	

Tree files from GARD output.

```
BEGIN TREES;

	TREE TREE_1 = (((((((NSPDE1AHAP1:0,NSPFR8BHAP19:0.00378083):0.00377484,NSPIT2AHAP23:0):0.00377494,NSPFR1AHAP13:0):0.00378307,(NSPDE1BHAP2:0.00376653,(((((NSPDE2AHAP3:0.00376881,(((NSPDE3AHAP4:0,(NSPDE3BHAP5:0.00376582,(NSPFR4AHAP15:0.00377508,((NSPIT5AHAP21:0,NSPIT9AHAP30:0):0.00376926,(NSPUS2AHAP32:0,NSPUS5AHAP35:0):0.00756519):0):0):0):0,NSPDE4AHAP6:0):0,NSPFR9AHAP20:0):0):0,((NSPFR10AHAP12:0,NSPFR8AHAP18:0.00376348):0,NSPIT1AHAP22:0.00376356):0.00376578):0,(NSPUS1BHAP31:0,NSPUS6AHAP36:0):0.00376884):0.00377725,NSPIT7AHAP28:0):0.00376592,NSPIT8BHAP29:0):0):0.00758442):0,(NSPIT3BHAP24:0,(NSPUS4AHAP33:0.00376583,NSPUS4BHAP34:0):0.00376856):0):0.00377177,(((NSPFR7AHAP16:0,NSPDE8BHAP11:0):0,NSPDE10AHAP7:0):0,NSPFR7BHAP17:0.0037702):0.00377248):0,((((((NSPIT4AHAP14:0,NSPDE8AHAP10:0.00377238):0,NSPFR6BHAP8:0):0,NSPDE5BHAP9:0):0,NSPIT10AHAP25:0):0,NSPIT6AHAP26:0):0,NSPIT6BHAP27:0):0,NSPUS7AHAP37:0);

	TREE TREE_2 = ((((((((((((((((((((NSPDE1AHAP1:0,NSPDE4AHAP6:0.00917503):0,NSPDE1BHAP2:0):0,NSPDE3AHAP4:0):0,NSPDE3BHAP5:0):0,NSPIT5AHAP21:0):0,NSPFR1AHAP13:0):0,NSPIT10AHAP25:0):0,NSPUS1BHAP31:0):0,NSPUS2AHAP32:0):0,((NSPDE2AHAP3:0.00612017,NSPUS7AHAP37:0):0,NSPFR8AHAP18:0.00305123):0.00305737):0.00611607,(((NSPIT6AHAP26:0.00305019,NSPIT6BHAP27:0):0,NSPIT8BHAP29:0):0.00305215,NSPIT9AHAP30:0):0):0.00612786,(NSPDE5BHAP9:0,(NSPUS5AHAP35:0,NSPUS6AHAP36:0):0.00305498):0):0.00305898,NSPIT4AHAP14:0.00305255):0,NSPFR10AHAP12:0):0,NSPFR9AHAP20:0):0,NSPIT2AHAP23:0):0,NSPFR7BHAP17:0):0,NSPUS4AHAP33:0):0,((((NSPFR4AHAP15:0,NSPIT7AHAP28:0.00305311):0,NSPFR8BHAP19:0):0,NSPFR6BHAP8:0):0,NSPDE8BHAP11:0):0):0,(((NSPIT1AHAP22:0,(NSPFR7AHAP16:0.00305114,NSPDE10AHAP7:0.00305258):0):0,NSPIT3BHAP24:0):0,NSPDE8AHAP10:0):0,NSPUS4BHAP34:0);

END;

BEGIN ASSUMPTIONS;

	CHARSET SPAN_1 = 1-264;

	CHARSET SPAN_2 = 265-594;

END
```

n	Contigs ID # (P. rapae, P. brassicae)	bp	S	dS	dN	dN/dS
1	1086, 391	387	11	0.2103	0.0002	0.001
2	1128, 628	222	7	0.2408	0.0002	0.001
3	1144, 530	531	71	0.2813	0.1067	0.3793
4	1187, 288	504	36	0.1961	0.0351	0.1792
5	1193, 513	432	14	0.1259	0.0001	0.001
6	1215, 638	747	56	0.3464	0.0126	0.0365
7	124, 381	609	22	0.1826	0.0042	0.023
8	1268, 742	426	34	0.2595	0.0356	0.1373
9	1289, 655	699	52	0.2613	0.0299	0.1142
10	1304, 815	504	22	0.127	0.017	0.1341
11	1320, 717	522	24	0.165	0.0133	0.0806
12	1401, 842	654	40	0.1235	0.0444	0.3597
13	1418, 388	735	46	0.2769	0.0146	0.0528
14	1428, 648	381	13	0.1304	0.0071	0.0548
15	1434, 833	381	17	0.1699	0.0074	0.0433
16	1452, 721	528	16	0.143	0.0001	0.001
17	1463, 104	699	50	0.2102	0.0351	0.1667
18	1464, 105	690	50	0.1661	0.0428	0.2576
19	148, 307	183	8	0.1941	0.0075	0.0385
20	1531, 858	744	43	0.1732	0.0292	0.1684
21	1544, 489	480	22	0.1521	0.012	0.0789
22	1583, 428	582	27	0.1995	0.0133	0.0669
23	160, 588	273	6	0.1001	0.0046	0.0462
24	1620, 856	459	30	0.2372	0.0288	0.1215
25	1633, 743	378	29	0.308	0.0251	0.0815
26	1655, 860	642	25	0.1507	0.0002	0.001
27	166, 762	582	19	0.2157	0.0041	0.0191
28	1665, 672	621	46	0.2596	0.0281	0.1084
29	167, 722	213	20	0.4365	0.0375	0.0859
30	1692, 456	510	16	0.0987	0.0109	0.1101
31	1727, 184	306	21	0.3263	0.013	0.0398
32	1733, 462	792	24	0.1185	0.0034	0.0288
33	1763, 788	645	34	0.1885	0.013	0.0688
34	1777, 309	459	42	0.1981	0.0733	0.3702
35	1801, 486	510	11	0.1024	0.0025	0.0244
36	1843, 632	441	41	0.3885	0.0306	0.0789
37	1878, 744	618	30	0.1777	0.0091	0.051
38	190, 745	642	42	0.254	0.0247	0.0973
39	2006, 540	468	30	0.2272	0.0204	0.0899
40	2034, 469	519	40	0.1905	0.0421	0.2212
41	2048, 501	612	17	0.1306	0.0001	0.001
42	2063, 586	195	5	0.0964	0.0127	0.1313
43	2115, 449	417	16	0.1278	0.013	0.1017
44	2116, 416	444	9	0.0797	0.003	0.0379
45	2169, 577	567	23	0.1436	0.0139	0.0968
46	2199, 493	660	29	0.1892	0.0041	0.0217
47	2201, 385	657	31	0.1759	0.0125	0.071
48	2205, 594	603	26	0.172	0.0067	0.039
49	2217, 832	300	16	0.1899	0.0097	0.0508
50	2344, 756	510	19	0.1711	0.0128	0.0749
51	2423, 673	324	16	0.1722	0.0045	0.0263
52	2427, 432	513	11	0.046	0.0086	0.1863
53	2437, 662	483	25	0.2017	0.0087	0.0429
54	2469, 657	408	8	0.0715	0.0001	0.001
55	2521, 306	675	26	0.1267	0.016	0.1265
56	2524, 791	474	36	0.2225	0.0429	0.1928
57	2525, 523	459	32	0.2438	0.0154	0.0633
58	2535, 374	597	36	0.152	0.0338	0.2224
59	2536, 630	489	19	0.2082	0.0026	0.0124
60	2554, 619	417	27	0.1548	0.0445	0.2875
61	2576, 866	519	31	0.1536	0.0329	0.2144
62	2585, 701	705	28	0.1549	0.0095	0.0615
63	2588, 490	627	26	0.2347	0.0079	0.0337
64	2593, 409	636	31	0.2075	0.0043	0.0206
65	2611, 620	714	19	0.0993	0.0001	0.001
66	2615, 355	750	46	0.1751	0.033	0.1888
67	2621, 477	600	29	0.2359	0.0146	0.062
68	2630, 526	372	25	0.157	0.0425	0.2706
69	2667, 634	486	34	0.3084	0.0139	0.045
70	2670, 775	534	19	0.1326	0.0145	0.1092
